# Supplementary material for: Reverse-Transcription Loop-Mediated Isothermal Amplification Has High Accuracy for Detecting Severe Acute Respiratory Syndrome Coronavirus 2 in Saliva and Nasopharyngeal/Oropharyngeal Swabs from Asymptomatic and Symptomatic Individuals
Source: J Mol Diagn. 2022 Apr;24(4):320–36. doi: 10.1016/j.jmoldx.2021.12.007 (PMC8806713; doi:10.1016/j.jmoldx.2021.12.007)
Supplement: Supplemental Table S1 [file mmc1.docx]

**Supplemental Table S1:** Serial dilution of Patient VTM (C_T_ = 19) 1:1 VTM into Lysis Buffer and 98°C heat treatment with and without heat pre-treatment at 56°C for 10 or 30 minutes.

| **P07553 (C_T_ = 19)** | **1:2** | **1:4** | **1:8** | **1:16** | **1:36** | **1:64** | **1:128** | **1:256** | **1:512** | **1:1024** | **1:2048** |
| --- | --- | --- | --- | --- | --- | --- | --- | --- | --- | --- | --- |
| **VTM 1:1 into Lysis + 98^°^C** | D | D | D | D | D | D | D | D | D | D | D |
|  | D | D | D | D | D | D | D | D | D | D | D |
| **56^°^C 10 mins pre-treat 1:1 VTM into lysis +98^°^C** | D | D | D | D | D | D | D | D | D | D | ND |
|  | D | D | D | D | D | D | D | D | D | D | ND |
| **56^°^C 30 mins pre-treat 1:1 VTM into lysis +98^°^C** | D | D | D | D | D | D | D | ND | D | ND | ND |
|  | D | D | D | D | D | D | D | D | ND | ND | ND |

D – RNA Detected, ND – RNA Not Detected, by Direct RT-LAMP.
