# Supplementary material for: Reverse-Transcription Loop-Mediated Isothermal Amplification Has High Accuracy for Detecting Severe Acute Respiratory Syndrome Coronavirus 2 in Saliva and Nasopharyngeal/Oropharyngeal Swabs from Asymptomatic and Symptomatic Individuals
Source: J Mol Diagn. 2022 Apr;24(4):320–36. doi: 10.1016/j.jmoldx.2021.12.007 (PMC8806713; doi:10.1016/j.jmoldx.2021.12.007)
Supplement: Supplemental Table S2 [file mmc2.docx]

**Supplemental Table S2 -** Serial dilution of Patient VTM (C_T_ 19.00 to 32.08) 1:1 VTM into Lysis Buffer and 98°C heat treatment with and without heat pre-treatment at 56°C for 10 or 30 minutes.

| **Patient VTM** | **P07553 (C_T_ 19)** | **P01127 (C_T_ 23.97)** | **P07102 (C_T_ 32.08)** | **P07392 (C_T_ 24.55)** | **P01071 (C_T_ 20.54)** |
| --- | --- | --- | --- | --- | --- |
| VTM into 1:1 Lysis + 98^°^C | D | D | D | D | D |
|  | D | D | D | D | D |
| 56^°^C 30 mins pre-treat 1:1 VTM into lysis + 98^°^C | D | D | ND | D | D |
|  | D | D | ND | D | D |

D – RNA Detected, ND – RNA Not Detected, by Direct RT-LAMP.
