# Supplementary material for: Reverse-Transcription Loop-Mediated Isothermal Amplification Has High Accuracy for Detecting Severe Acute Respiratory Syndrome Coronavirus 2 in Saliva and Nasopharyngeal/Oropharyngeal Swabs from Asymptomatic and Symptomatic Individuals
Source: J Mol Diagn. 2022 Apr;24(4):320–36. doi: 10.1016/j.jmoldx.2021.12.007 (PMC8806713; doi:10.1016/j.jmoldx.2021.12.007)
Supplement: Supplemental Table S3 [file mmc3.docx]

**Supplemental Table S3 -** Sensitivity of the viral culture assay – 1 PFU/mL

| Number of PFU added to AVL/flask | E Gene RT-PCR assay | | | Growth (CPE) | |
| --- | --- | --- | --- | --- | --- |
|  | C_T_ value of dilution (AVL) | Baseline Mean C_T_ value from flasks | Final Mean C_T_ value from flasks | Flask 1 | Flask 2 |
| 1000 | 19.64 | 25.0 | 12.2 | + | + |
| 100 | 22.65 | 28.4 | 11.8 | + | + |
| 10 | 26.00 | 31.8 | 11.9 | + | + |
| 1 | 28.73 | 35.0 | 12.6 | + | + |
| 0.1 | 32.11 | 37.6 | 37.3 | - | - |
| 0.01 | 34.43 | 39.8 | 37.7 | - | - |
