# Supplementary material for: Reverse-Transcription Loop-Mediated Isothermal Amplification Has High Accuracy for Detecting Severe Acute Respiratory Syndrome Coronavirus 2 in Saliva and Nasopharyngeal/Oropharyngeal Swabs from Asymptomatic and Symptomatic Individuals
Source: J Mol Diagn. 2022 Apr;24(4):320–36. doi: 10.1016/j.jmoldx.2021.12.007 (PMC8806713; doi:10.1016/j.jmoldx.2021.12.007)
Supplement: Supplemental Table S4 [file mmc4.docx]

**Supplemental Table S4** - RT-LAMP results of time course from symptom onset

| Days post suspected exposure | Direct RT-LAMP | | | RNA RT-LAMP | | | RT-qPCR C_T_ | | Observed Symptoms |
| --- | --- | --- | --- | --- | --- | --- | --- | --- | --- |
|  | Tp 1 | Tp 2 | Result | Tp 1 | Tp 2 | Result | ORF1ab | N |  |
| 5 | 09:18 | 09:32 | POS | 11:09 | 10:50 | POS | 23.31 | 26.47 | Onset: Sore throat. Blocked nose. Headache. Lack of appetite. Fever. |
| 6 | 10:32 | 11:34 | POS | 10:34 | 10:40 | POS | 21.16 | 24.03 | Sore throat. Headache. Restless sleeping. Tired |
| 7 | 10:19 | 13:40 | POS | 11:39 | 09:59 | POS | 24.47 | 27.30 | Headache. Restless sleeping. Tired. Loss of smell and taste. |
| 9 | 09:31 | 09:14 | POS | 08:35 | 09:37 | POS | 28.55 | 31.89 | Tired. Loss of smell and taste. |
| 11 | 13:14 | 11:47 | POS | 16:44 | 16:39 | POS | 26.44 | 29.06 | Tired. Loss of smell and taste. |
| 12 | 09:10 | 10:09 | POS | 12:42 | 12:01 | POS | 26.13 | 29.19 | Tired. Improvement in smell and taste. |
| 13 | NEG | NEG | NEG | 14:06 | 12:56 | POS | 28.16 | 30.62 | Significant improvement in all symptoms |
| 14 | NEG | NEG | NEG | NEG | NEG | NEG | 38.05 | 40.73 | None |
| 16 | NEG | NEG | NEG | NEG | NEG | NEG | 36.11 | NEG | None |
| 17 | NEG | NEG | NEG | NEG | NEG | NEG | NEG | NEG | None |

Time to positivity in minutes [Tp]; Cycle Threshold [C_T_]; Negative [NEG]; positive [POS]
